# Opioid Use in Pregnant Women and Neonatal Abstinence Syndrome—A Review of the Literature

**DOI:** 10.3390/toxics7010009

**Published:** 2019-02-16

**Authors:** Fábio Martins, David Oppolzer, Catarina Santos, Mário Barroso, Eugenia Gallardo

**Affiliations:** 1 Centro de Investigação em Ciências da Saúde, Faculdade de Ciências da Saúde da Universidade da Beira Interior (CICS-UBI), 6200-506 Covilhã, Portugal; fabiomartins23@hotmail.com (F.M.); davidmarkl9@gmail.com (D.O.); katherijn@gmail.com (C.S.); 2 Serviço de Química e Toxicologia Forenses, Instituto de Medicina Legal e Ciências Forenses—Delegação do Sul, 1150-334 Lisboa, Portugal; mario.j.barroso@inmlcf.mj.pt; 3 Laboratório de Fármaco-Toxicologia—UBIMedical, Universidade da Beira Interior, 6200-284 Covilhã, Portugal

**Keywords:** opiates, in utero drug exposure, pregnancy, neonatal abstinence syndrome

## Abstract

Opiate use during pregnancy has been an increasing problem over the last two decades, making it an important social and health concern. The use of such substances may have serious negative outcomes in the newborn, and clinical and cognitive conditions have been reported, including neonatal abstinence syndrome, developmental problems, and lower cognitive performance. These conditions are common when opiates are used during pregnancy, making the prescription of these kinds of drugs problematic. Moreover, the mother may develop opiate addiction, thus, increasing the likelihood of the infant being born with any of those conditions. This paper reviews the use of opiates during pregnancy and focuses mainly on the neonatal abstinence syndrome. First, the commonly prescribed opiates will be identified, namely those usually involved in cases of addiction and/or neonatal abstinence syndrome. Second, published approaches to deal with those problems will be presented and discussed, including the treatment of both the mother and the infant. Finally, we will outline the treatments that are safest and most efficient, and will define future goals, approaches, and research directions for the scientific community regarding this problem.

## 1. Introduction

Opioids are narcotic drugs that act on opioid receptors. This results in an analgesic effect, and these compounds are used worldwide mainly for the treatment of acute and chronic pain. However, in the last two decades, the incidence of opioid prescriptions has had an alarming increase. Indeed, in a study performed in Canada between 2006 and 2011, Gomes et al. [1] reported an increase of 23% in the prescription of high-dose opioids. A much higher increase was reported in the USA between 2000 and 2010, where opioid prescriptions have increased by 104% [2].

Data from the Centers for Disease Control (CDC), concerning the general population of the 50 states and the District of Columbia of the United States of America, shows a similar incidence, with an increasing number of opioid prescriptions between 2006 and 2012. The figures concerning prescription rates have peaked in 2012, with 81.3 opioid prescriptions per 100 patients. While the number of cases has lowered substantially by 2017, the rate of 58.7 opioid prescriptions observed in this year is still of concern [3]. Data collection was based on a sample of approximately 50,000 retail pharmacies, which dispense around 90% of all retail prescriptions in the United States. Cough and cold medications containing opioids and buprenorphine products typically used to treat opioid use disorders were not included. Methadone dispensed under the scope of maintenance treatment programs was not included as well.

This increase was not exclusive to North America, as an increase in opioid consumption was also observed in Denmark, Norway, Finland, Sweden, and Iceland [4]. Guidelines have been published by CDC to specify those situations where opioid prescription is recommended, and identifying indirect problems arising from the actual clinical practice regarding the use of these drugs. Contrary to current clinical practice, opioid drugs should only be used in situations where it is proven necessary, and specially only after all other options have been explored and discarded for lack of efficiency [5]. Alternative approaches should be used whenever possible, for instance using acetaminophen or nonsteroidal anti-inflammatory drugs (NSAIDs), as these represent safer options that have a low risk of addiction problems compared to opioids. In addition, these alternative approaches have also been proven to be very useful in the treatment of pain [6]. Nevertheless, these alternative compounds are usually not considered as first line agents.

Pregnant women are a critical population in what concerns the use of opioids, due to the severe effects and consequences of opioid use both for the mother and the infant. While addiction is the main problem regarding pregnant women who take these drugs, we must consider the fact that all opioids cross the placenta and are capable of reaching the infant; therefore, the effects of their use must be also considered in the infant, since this can make the difference between death or survival [7]. Patrick et al. [8] reported an alarming figure, that 28% of pregnant women confirmed having filled one or more opioid prescriptions during their pregnancy. The consumption of opioids during pregnancy is a serious public health problem worldwide; therefore, in order to account for the deleterious effects in newborns, as well as the social and legal consequences of the abuse of these substances, it is important to have insight on the published research on the matter, and also on the approaches to deal with these situations. In addition, and to avoid misleading the reader, we have used the term “opioids” throughout this review, since opiates (not only the naturally occurring alkaloids morphine and codeine, but also semisynthetic derivatives such as heroin, methadone, fentanyl, hydromorphone and buprenorphine) may also be included under this definition.

In order to make this review more readable and easier to follow, a brief introduction on opioid use during pregnancy will be presented. Neonatal abstinence syndrome, including diagnosis and treatment-related aspects, will be discussed thereafter.

## 2. Research Methodology

A literature search was performed using the PubMed database, Cochrane Library, and Google Scholar. For both PubMed and Google Scholar databases, the following search strings were used: “neonatal abstinence syndrome”, “opioid use pregnancy”, “neonatal abstinence syndrome treatment”, “opioid addiction in pregnancy treatment”, and “drugs in utero” combined with Boolean operators. Also, a search with each of the opioids described in this paper was done combined with the terms “neonatal abstinence syndrome treatment” or “pregnancy addiction treatment”. For the Cochrane Library, systematic reviews were searched with the terms “neonatal abstinence syndrome treatment” and “opioid pregnancy treatment”. This search occurred between September and December of 2018. No publishing date restrictions were used. All articles relevant to the subject were considered as long as they were written in English. In order to assess their relevance, all papers fulfilling the search strings were screened independently by four of the authors. Only those that were selected by at least two authors were subjected to review and were included in the manuscript.

## 3. Opioids Use during Pregnancy

In a cohort study performed in 46 states of the United States of America, it was verified that opioids such as codeine, oxycodone, hydrocodone, and propoxyphene were the most prescribed compounds, and as such could be considered responsible for most of the neonatal abstinence syndrome (NAS) cases [9]. Other opioids such as methadone and buprenorphine, which are usually prescribed to opioid dependent patients as a way of treatment, have also been associated with NAS cases, although conclusions regarding a direct correlation between buprenorphine/methadone and NAS may be misleading since these cases involved women that were already opioid dependent before treatment [10]. Nonetheless, buprenorphine and methadone rightfully have the addiction label attached to them and are known to cause withdrawal symptoms in the infant [10,11]. A study from 1973 reported that methadone-related withdrawal effects in infants were more aggressive than those of heroin [12]. Another opioid capable of causing withdrawal symptoms is fentanyl. Regan et al. [13] reported a case of NAS following prolonged fentanyl administration during pregnancy. In this case, the pregnant woman reported suffering from soft tissue injuries in her cervical and lumbar spine. Alternative treatment approaches, such as nonsteroidal anti-inflammatory drugs, anti-spasmodics, and physiotherapy, had failed in controlling the pain, and as such she was being treated with transdermal fentanyl. 

Codeine may be considered an outlier prescription opioid because it is used mostly as an antitussive, despite its use as an analgesic in combination with other drugs such as acetaminophen. It is perceived as a safer option in comparison to all other opioid drugs. However, despite the fact that it is less potent and addictive than other opioids, it is known that codeine is metabolized into morphine in humans. Codeine is often prescribed because it is a much less controlled drug, increasing the risk for its abuse during pregnancy. Van Leeuwen et al. [14] have reported the first case of NAS following codeine usage in 1965. In addition, the combination of paracetamol and codeine was reported in 1997, and these being the only drugs used during pregnancy leads to a high probability that they caused NAS [15]. Contrary to these reports, a large population-based cohort study from 2011 reported no correlation between codeine intake during pregnancy and neonatal survival rate, congenital malformation rate, or any other adverse outcomes. The study states however that an association between codeine and NAS was not directly studied, despite the fact that low Apgar scores and no admission into intensive care may be an indirect factor leading to the conclusion that these two do not correlate [16]. Nonetheless, in 2005, death of a breastfed infant where the mother was taking codeine was reported, with this substance being identified as the most probable cause of death [17]. Infants may metabolize codeine less efficiently than adults, and this increases their exposure to the drug and the risk of NAS [18]. Codeine does not seem to be a high-risk opioid in terms of NAS, but care should be taken when prescribing this drug to pregnant women since that risk, albeit low, exists.

Heroin, which is mostly used as an illicit drug for recreational use, is a morphine derivative responsible for a large number of NAS cases. It is a highly addictive drug; therefore, addicted women will likely continue using this drug throughout pregnancy, resulting in NAS in most of the cases [19]. Furthermore, even if its use is stopped, a period of substitution treatment with buprenorphine or methadone follows, exposing the infant to them throughout pregnancy [10].

Lastly, in a more recent report, a NAS case was reported due to the use of *Mitragyna speciosa* during pregnancy, a plant commonly known as Kratom that is used and perceived as a safer option than opioids for addicts [20]. This plant, however, causes withdrawal symptoms and craving in users [21], leading to the report of the newborn girl showing NAS symptoms [20].

## 4. Neonatal Abstinence Syndrome

The different effects of opioids on users have been known for several years. Their addictive potential is one of the major reasons for the current control of these drugs. Consequently, a woman addicted to opioids is likely to continue using these substances during pregnancy, as mentioned previously. Moreover, as discussed in the last section, opioids have been increasingly prescribed to pregnant woman, resulting in risk exposure to the infant that is often neglected. Not only are opioids known for their addiction problems, but also for the severe withdrawal symptoms associated with stopping their use, which may occur both in the mother and newborn. Withdrawal symptoms in the infant appear as a consequence of prolonged exposure to these addictive substances during gestation, since they cross the placenta barrier [7]. After discontinuation of opioid exposure, abstinence symptoms start appearing and are generally described as NAS. As discussed previously, the number of NAS cases is increasing with the increase in opioid prescriptions. As an example, in a recent study made in West Virginia, approximately 5% (53 NAS cases in 1000 live births per year) of NAS cases have been reported [22]. 

Although the mechanisms behind this syndrome are not totally clear, it is known that there is an increase in the release of neurotransmitters (dopamine, acetylcholine, norepinephrine, serotonin, and corticotrophin). This consequently leads to numerous identifiable symptoms in the infant, as outlined in Table 1 [23]. The information presented in Table 1 is crucial in establishing a diagnosis in the newborn as soon as possible. These symptoms develop from 24 to 72 h post-partum, although in some exceptional cases they may appear up to 10 days after birth [19].

## 5. Neonatal Abstinence Syndrome—Diagnosis and Evaluation 

Since a high number of symptoms are usually associated with NAS, and many of them can originate in different and/or impaired diagnoses, toxicological confirmation should often be performed [24]. 

### 5.1. Medical Scoring Tools

One way to obtain a correct diagnosis without using toxicological analysis is by means of scoring systems, namely the Finnegan Scoring Tool [25], which was created in 1975 and designed for NAS diagnosis [26]. This is one important tool that helps health professionals throughout both diagnosis and treatment. Zimmermann-Baer et al. [27] presented a modified version of this tool (Table 2). Health professionals taking care of the newborn usually evaluate if any of the symptoms in Table 2 are present and add the scores at the end. Neonatal abstinence syndrome is highly likely to be the diagnosis if the sum of scores is higher than 8. If a score of 9 or higher is obtained in two separate evaluations, pharmacological treatment should be readily initiated. Another useful tool to evaluate neonate symptoms is the Apgar score [28], a test that is performed on the infant at the first and fifth minute post-partum. The first test gives information on how well the neonate tolerated partum, while the second test gives information on how well the baby is doing. This test evaluates breathing effort, heart rate, muscle tone, reflexes, and skin color; this correlates to the symptoms in Table 2, and is of value for the Finnegan score tool if used too. Recently Grossman et al. [29] proposed a novel approach to assess infants with NAS. This approach consisted of an Eat, Sleep, Console (ESC) management, instead of the traditional Finnegan Neonatal Abstinence Scoring System (FNASS). This ESC approach was considered effective in infant management if the infant was breastfed well or was able to eat ≥1 oz per feed, was able to sleep undisturbed for more than 1 h, and was able to be consoled within 10 min after the onset of crying. The authors concluded that infants who were managed using the ESC approach were treated with morphine significantly less frequently than they would have been if the Finnegan score had been used.

These are methods of diagnosis applied to the neonate; however, focusing on the pregnant woman may also be a reliable way of predicting and/or diagnosing NAS as well. For instance, the use of heroin is an indicator of high probability of NAS, as 48% to 94% of infants born with NAS were reported in women taking heroin or methadone during pregnancy [19]. One of the most important factors and prevention measures that should be taken to avoid NAS in the newborn is improving the mother’s awareness of the consequences of using opioids during pregnancy, may they be illicit or not. Indeed, the increase in the use of prescription opioids during pregnancy was associated with the increase in NAS cases [22]. 

### 5.2. Analysis of Biological Specimens

Despite the usefulness of observational methods of diagnosis, such as the above described scoring tools, a more concrete way of predicting and/or diagnosing this pathology may be the analysis of biosamples from the mother or the newborn.

Urine or saliva tests can be used throughout pregnancy to obtain information on whether the opioids are being used or not [19], and this may be a very important measure to take when women are at risk of opioid consumption. This refers mostly to known drug addicts, as sporadic opioid consumption is usually unknown to the health professionals, which means that only by regular routine tests can NAS be predicted and/or prevented. Nonetheless, prescription opioids are a major factor that must be taken into account when dealing with NAS diagnosis and prevention. Although it may be thought that most NAS cases are associated with illicit opioid abuse, 65% of infants born with NAS were reported to have been exposed to at least one prescription opioid during pregnancy in a cohort study published in 2015 [8].

The identification and quantification of drug biomarkers in both the maternal and newborn samples offer objective evidence of exposure. Each biological matrix has pros and cons, and presents individual variability in terms of windows of detection and specific drugs and metabolites present. Among the possible biological samples from the mother (urine, blood/plasma/serum, oral fluid, sweat, nails, and hair), the newborn (urine, hair, nails, and meconium), and the maternal-fetal unit (amniotic fluid, placenta, and umbilical cord), maternal hair usually presents the window of detection for drugs during the whole pregnancy, meconium is the current gold standard that allows detecting drug exposure from the third trimester, and the umbilical cord is the emerging alternative matrix to meconium. The monitoring of maternal biological specimens provides important information for the clinician in order to facilitate a prompt intervention and attempts to reduce the frequency and magnitude of drug exposure [30,31,32]. Moreover, the identification of in utero drug exposure may also have legal consequences, namely as evidence of child abuse. On the following lines, the advantages and drawbacks of each of these biological specimens will be discussed in regard to in utero drug exposure assessment.

Blood/plasma/serum and oral fluid have short windows of detection, from several hours to days, depending on the drug, dose, and route of administration. In the particular case of opioids, this window is approximately 12–24 h. However, oral fluid offers several advantages when compared to the classical biological samples, namely its easy and non-invasive collection procedure, its low biohazard risks, and its ability to allow direct observation of the individual, thereby reducing the opportunity for sample adulteration or substitution. Disadvantages of oral fluid testing include little sample availability and very low concentrations for some analytes [32,33,34]. Enders and McIntire [35] used 0.1 mL of sample and detected 8 opioids and metabolites, while Di Rago et al. [36], Montesano et al. [37], and Liu et al. [38] have published multi-analyte methods, identifying from 10 to40 abused drugs in oral fluid specimens. Urine testing for abused drugs is performed in laboratories worldwide, and urinalysis nowadays includes well-established and well-known methods. However, drug testing of urine specimens presents some drawbacks, and the real possibility of sample adulteration or substitution is of concern [32,39]. As a consequence, urine samples are often tested for authenticity. In addition, and since morphine is excreted in urine mainly as the inactive metabolite morphine 3-glucuronide, testing urine for opiates usually begins with hydrolysis of the conjugate to liberate free morphine prior to sample cleanup and chromatographic analysis [33]. Sweat is another alternative specimen that is mainly applied in workplace drug testing scenarios and in monitoring drug exposure [32,39]. In general, sweat patches are applied to the skin for seven days, and the excreted drugs accumulate in sweat over the entire wear period. Perhaps the main problem of this sample is the difficulty in estimating sweat volume and in evaluating proper drug concentrations [31,32]. Due to these limitations, the number of publications dedicated to the detection of opiates in this particular matrix is scarce [40,41,42]. In the case of biological samples from the newborn, collection is difficult because neonatal blood/plasma/serum collection procedures are highly invasive, and skin irritation from the adhesive on the urine collection bag can occur, which also frequently fails to adhere, resulting in sample loss.

Other alternative samples useful to monitor drug exposure are nails and hair. Nails are a keratinized biological matrix that can accumulate drugs over time. Fingernails grow at an average rate of 3 mm/month (1.9–4.4 mm/month), while toenails grow at a 30–50% slower rate. As nails grow, drugs and metabolites incorporate into the keratin fibers for 3–5 months in fingernails, and 8–14 months in toenails. Nails allow for constant monitoring of drug consumption, but there is little controlled drug administration data to aid interpretation of drug concentrations in nails [43,44,45]. There are many endocrine and metabolic changes in pregnant women that may produce changes in nails. Erpolat et al. [46] reported that the most common nail pathology was leukonychia (24.4%), increasing up to 27.4% in gestational weeks 29–42. Leukonychia affects keratin fiber formation and maturation, and it is not known how this might affect drug and metabolite incorporation into nails. Neonatal nail collection is difficult because all nail clippings in the first 3 months are needed and these samples are not always continuously collected by the mother [47]. One of the most important limitations is related to analytical procedures. Nail analysis requires efficient washing of the sample to eliminate external contamination, pulverization, digestion to extract drugs and metabolites from the keratin fibers, and extraction to further isolate and concentrate substances [30,48,49]. Shen et al. [50] described a method capable of detecting morphine, acetylcodeine, codeine, heroin, and 6-acetylmorphine in 20 mg samples of nails. Cappelle et al. [51] proposed a multimethod that analyses 12 drugs using 20 mg of sample as well. 

Hair is one of the alternative samples most used for this purpose. Hair has the longest window of detection compared to other specimens, depending on its length. Neonatal hair begins to form during the second trimester, around 17–20 weeks of gestational age, so hair is also a keratinized matrix that accumulates drugs/metabolites over time; for this reason, it allows drug monitoring from the third trimester on. Possible routes of drug incorporation into hair include diffusion from blood, sweat and sebum, and from the environment (external contamination). Hair grows on average at a rate of 1 cm/month, with the first centimeter of hair proximal to the scalp reflecting exposure over the last month, and as one proceeds outwards, older exposure situations can be identified. Each hair follicle has three stages of growth: active growth (anagen), involution (catagen) and rest (telogen) [33,52,53,54]. Drugs and metabolites are incorporated from blood into the hair during the anagen phase. The posterior vertex region of the head has the highest percentage of hair follicles in the growing phase (85% anagen phase, 15% catagen and telogen phase). Although during the second and third trimesters of pregnancy a higher percentage of hair follicles are in the anagen phase (95%), the mean hair growth rate remains 1 cm/month. With collection of hair specimens shortly after delivery, the 3 cm segment closest to the scalp reflects the third trimester, the segment >3–6 cm from the scalp reflects the second trimester, and the segment >6–9 cm from the scalp reflects the first trimester of pregnancy [30,55]. Like neonatal nails, neonatal hair is exposed in utero to amniotic fluid, which affects the incorporation amount and timing of drugs and their metabolites. Neonatal hair samples are often not available, at least not in sufficient amount, and many parents refuse its collection because of cosmetic or cultural reasons [56]. In order to help in performing the analysis, the guidelines from the Society of Hair Testing (SoHT) [57] recommend cutoffs for substances and metabolites in hair to identify active use. The number of analytical methods available for the determination of opiates in hair samples is very wide [58,59,60,61,62]. Meconium is the gold standard in assessing in utero drug exposure because of its wide detection window. Meconium is the first stool of an infant, usually passed in the first 72 h after birth, ranging from 20 to 70 g [32,63]. Meconium formation starts around the 12th week of gestation and accumulates until birth. About 1 g of meconium accumulates in the fetal gut during 23–26 weeks of gestation (end of the second trimester) and about 5 g after 27–32 weeks (beginning of the third trimester). Nearly 80% of meconium accumulates after 38 weeks of pregnancy [64]. Therefore, meconium analysis provides an overview of drug exposure primarily during the last trimester. The disadvantages of meconium testing include the sequential sample collections needed over time, requiring up to 5 days, or sample loss if meconium is passed in utero. Also, meconium can be contaminated by neonatal urine, and test positive for drugs administered during delivery [30,65]. Some publications recommend different procedures to detect opioids in these samples, for instance the recent works of Marin et al. [66], which detects buprenorphine and its metabolite in 0.25 g of meconium, or the work of Concheiro et al. [67], where these compounds are detected in several specimens including meconium. 

Other biosamples from the maternal-fetal unit are amniotic fluid, umbilical cord blood, umbilical cord tissue, and placenta. However, these specimens are not often used because of the absence of adequate studies, difficulties in sample collection (e.g., amniotic fluid), or the scarce data about the correlation between samples (e.g., cord blood and peripheral neonatal blood). For these reasons, the number of publications involving those specimens is still scarce.

These specimens present some advantages, namely concerning the windows of exposure, particularly in the case of the umbilical cord, where the window of detection is similar to that of meconium, reflecting drug exposure in the last trimester of pregnancy [30,32,63]. However, the composition, origin, and function of each of these matrices may affect the extent that specific drugs and metabolites are accumulated therein. For instance, in the case of opioids, free morphine is the primary analyte detected in meconium; however, in the umbilical cord, determining morphine-3-glucuronide is critical [68,69]. Currently, there are no officially recommended cutoffs or required analytes for meconium or umbilical cord testing to access in utero drug exposure [30]. Concerning analytical instrumentation, mass spectrometry (MS) or high-resolution instrumentation (HRMS) is mandatory due to their sensitivity in detecting vestigial concentrations found in the different specimens. Triple quadrupole LC–MS/MS systems, operated in the multiple reaction-monitoring mode, are becoming the reference analytical tool for identification and quantification of drug markers in biological samples due to their versatility, sensitivity, and specificity. Analytical methods must be validated and fulfill confirmation criteria, requiring a minimum of two transitions per compound and a ratio within certain established limits.

## 6. Opioid Addiction in Pregnancy—Treatment

Previously, substitution treatment in opiate-addicted pregnant women was unadvised [70]. However, in the present, it is recommended that these patients undergo opioid agonist pharmacotherapy [71]. Medically supervised withdrawal is a type of treatment that has little success, since with the appearance of withdrawal symptoms, these women usually relapse and go back to taking opioids. The percentage of relapse in these cases ranges from 59% to more than 90% [72], and consequently the pregnancy outcomes are worse. To support and highlight this recommendation, in a study performed by Bell et al. in 2016, 301 fully detoxified opioid-addicted pregnant women saw no fetal outcomes related to this same detoxification [70]. Nonetheless, in this study 31% of neonates were reported to have NAS at birth. Also, 36% of the treated women had relapsed, which accounted for most of the described NAS cases [70]. Not only does this suggest that treatment is crucial to prevent NAS, as the reported percentage is low enough to conclude that treatment is of high value in these situations, but it also suggests that treatment is not enough, concerning the significance of the occurred NAS cases Thirty-six percent of the total women relapsed after (or during) treatment. Therefore, treatment should go along with long-term behavioral health management after the patient is drug free, as suggested in the study, to improve outcomes and prevent relapses [70]. A review from the European Monitoring Centre for Drugs and Drug Addiction (EMCDDA) [73] also recommends psychologically assisted opioid substitution as a first line treatment for these patients. The most commonly used opioid agonists in the treatment of opioid dependence are methadone and buprenorphine [70,71,73]. In a retrospective cohort study conducted between 2000 and 2012, the treatment of 609 opioid addicted pregnant women was assessed. From these, 248 were treated with methadone and 361 with buprenorphine [74]. The dosages of these opioid agonists were adjusted based on the severity of symptoms or in the case of withdrawal symptoms. Methadone was the only drug used up until 2004, while the use of buprenorphine increased over time from 2004 to 2008 [74]. Moreover, adding to the fact that they were being treated with opioid agonists, counseling in the community by healthcare professionals was advised to all women receiving the treatment [74], in agreement to what is suggested by Bell et al. [70]. Conclusions taken by this cohort study indicated that pregnant women treated with buprenorphine have outcomes at least comparable to those of methadone, as well as a lower number of reported NAS cases in the group under treatment with buprenorphine [74]. A possible explanation is the fact that buprenorphine does not cross the placenta as efficiently as methadone, resulting in a lesser exposure of the fetus to the opioid drug and fewer NAS cases in this group [10,74]. Another study by Jones et al. also reported similar findings [10]. Despite this, it seems that buprenorphine is related to a higher incidence of relapsing [75]. Buprenorphine users attain a clearer state of mind with this opioid, and in search of reversing this status, relapse occurs [75,76]. However, Fischer et al. [77] reported that although retention rates were higher in the group treated with methadone, buprenorphine treated women had a significantly lower rate of opioid consumption, confirming that buprenorphine may be a better alternative to methadone in the treatment of opioid addicted pregnant women. Additionally, Tolia et al. [78] reported in a large retrospective cohort study published in 2018 that infant exposure to buprenorphine resulted in lesser pharmacological treatment for NAS and shorter periods of hospital stay when compared to methadone. The Maternal Opioid Treatment: Human Experimental Research (MOTHER) study, an eight-site randomized, double-blind, double-dummy, flexible-dosing, parallel-group clinical trial, shows little statistical difference between the methadone and buprenorphine treated groups [79]. In terms of treatment and transition from one drug to another, Johnson et al. [80] described a transition protocol to change from methadone therapy to buprenorphine, either due to unacceptability or ineffective treatment with the former. In that paper, the authors reported that more than 80% of women taking buprenorphine were satisfied with the treatment, and only one out of twenty women included in the study did not continue the treatment after the transition. 

Other pharmacological treatments include using naltrexone, a nonselective opioid antagonist used more commonly in non-pregnant opioid abusers to help their efforts in maintaining abstinence [71]. It is reported that although its oral dosage form does not have significantly better results than placebo, its long-acting injectable formula may have favorable results in the treatment of opioid addiction [71,81]. However, little information exists regarding its safety during pregnancy, and the risk of relapse is increased in these situations [81]. Lastly, naloxone is an opioid antagonist used to reverse effects of opioid overdose, and useful only in these cases in pregnant women [71].

## 7. Neonatal Abstinence Syndrome—Treatment

After the infant is born and NAS symptoms start to appear, a diagnosis must be made. In the case of a positive NAS diagnosis, appropriate treatment(s) should follow as soon as possible. The initial treatment should be primarily supportive, avoiding exposure of the infant to any unnecessary drugs with consequent risks [82]. In the case where this approach is not enough, and if the symptoms worsen, opioids should be the drugs of choice, as they have been first line therapy for over 40 years [83]. Using these compounds, the resolution of this syndrome is faster than with other options, with the advantage of alleviating some symptoms (e.g., reducing the frequency of seizures) [84]. Options such as opioid tincture and paregoric opium were used in the past, with morphine becoming more commonly used in these treatments since it has a fast titration and considerably reduces withdrawal symptoms [83]. The use of paregoric (camphorated tincture of opium) has been stopped, due to its high percentage of ethanol and other toxic excipients; the use of other drugs, such as naloxone, has stopped as well, due to the possibility of seizures. Benzodiazepines and chlorpromazine have been tested as well, but their use is not recommended because of their long half-life and possible adverse effects on the neonate [85]. Methadone and buprenorphine, similar to what is seen in the treatment of addicted pregnant women, are now the most common choices [84,86,87]. Methadone, in comparison to morphine treatment, was reported by Brown et al. [87] to have resulted in a shorter length of treatment. In their study, the authors observed that infants were on methadone treatment for 14 days, while with morphine it lasted for 21 days. In this randomized, double-masked trial, the results of methadone therapy were found to be overall better than those of morphine [87]. Despite the fact that both morphine and methadone therapy still remaining as the standard for NAS care, there are known adverse effects related to the use of both drugs. These effects pose a risk to users, namely in the case of significant respiratory depression and slowed gastrointestinal motility, which may lead to feeding intolerance [83]. This highlights the need to closely monitor the infant under treatment to be able to quickly detect and solve any problems that may arise. Safer treatment alternatives are being continuously researched, leading to a cohort study performed in 2015 where a comparison between a pilot buprenorphine therapy and methadone treatment for NAS was done [86]. In this study, a similar duration to the one reported by Brown et al. [87] was observed. The reported length of methadone treatment in this study was 14 days. In comparison, the duration of the treatment with buprenorphine was 9.4 days, considerably lower when compared to methadone. In addition to that, hospital stay was also slightly shorter with buprenorphine [86]. Moreover, no significant adverse effects were observed in any of the groups [86]. In another clinical trial, Kraft et al. reported a shorter length of hospital stay when comparing buprenorphine and morphine therapies for the treatment of NAS. A median duration of 15 days in the buprenorphine group compared to 28 days in the morphine group showed a comparable difference, similar to what was described previously in other cited studies [88]. What stands out in this clinical trial is the reference to a slight difference in respiratory rate between the two groups. The mean respiratory rate in the morphine group was 4.4 breaths per minute lower than buprenorphine, indicating that this last opioid agonist may be safer than morphine [88]. Despite all this, it is important to note that buprenorphine formulations usually have ethanol, which is highly unadvised to be administered to an infant [26].

Additionally, adjunctive therapy is commonly used to accelerate treatment and reduce the length of hospital stay. Options such as phenobarbital, clonidine, diazepam, and chlorpromazine were tested. As stated before, diazepam was reported to be associated with a number of concerning adverse effects as well as withdrawal symptoms after treatment [89], similarly to what occurs with chlorpromazine, which has been associated to cerebellar dysfunction as well as hematological problems [82]. In the case of clonidine, a randomized clinical trial performed between 2002 and 2005, where 80 infants were enrolled, showed a significant decrease (27%) in length of therapy (when compared to placebo), lower dosages of opioid drug needed for treatment, and no treatment failure in the clonidine group. Moreover, few adverse effects during hospital stay were reported, although 3 cases of death happened after hospital discharge and before 6 months of age, but were not related to treatment [90]. In 2013, a prospective randomized clinical trial compared both clonidine and phenobarbital as adjunctive therapy. Morphine sulfate was used as the main drug for therapy. When compared, phenobarbital had clinically shorter, but not significant, inpatient time, whereas clonidine had shorter overall therapy time, proving to be a viable alternative. Furthermore, little adverse effects were associated with clonidine, and no mortality was observed during the clinical trial [91]. In another study performed between 2003 and 2006, additional data favorable to the use of clonidine was reported. However, in this case, the drug was used as main prevention and treatment therapy in 14 infants born from opioid dependent mothers [92]. While this study was performed in a very small population, it corroborates the fact that clonidine use is of value in the treatment of NAS. In the case of phenobarbital, Osborn et al. reported in their review paper that phenobarbital has been shown to be better in supportive therapy in the treatment of NAS, as the reduction of symptoms and the prevention of treatment failure is higher when using this drug. However, and as the authors concluded, phenobarbital has not been shown to be superior to opioids [84]. Nonetheless, as adjunctive therapy phenobarbital has its value as seen in the study where it was compared to clonidine, and the former was seen to reduce inpatient time [91]. Moreover, in a study performed by Coyle at al. [93] phenobarbital was used together with diluted tincture of opium (DTO), and the results were compared to those obtained with DTO alone. Treatment with phenobarbital was reported to have reduced length of stay by 48%, as well as shorter prevalence time of severe withdrawal symptoms.

Finally, in a more recent study, laser acupuncture was used as adjunctive therapy to oral morphine. Raith et al. [94] published a study where this kind of therapy was used in 28 newborns, according to a predefined protocol so that every infant had the same treatment and procedures, as well as the same acupuncture points. The duration of the acupuncture therapy was the same as the oral morphine. A significant reduction in the duration treatment between the group treated with laser acupuncture and the control group was observed (20 versus 39 days) as well as length of hospital stay (35 versus 50 days). This therapy was well tolerated, no adverse effects were observed, nor negative feedback from parents or hospital staff was reported.

## 8. Healthcare Professionals’ Attitudes

First, and as pointed out in the beginning of this review, the opioid epidemic seen today is a consequence of an exceeding number of prescriptions of these drugs. Together with heroin consumption by pregnant women, this has led to an increase in NAS cases over the last two decades. Changes need to be made in terms of prescription, and healthcare professionals need to be aware of the risks of prescribing opioids to pregnant women. Also, these women need to be informed of the possible consequences that the prolonged use of these drugs can have in their infant. Even codeine, often regarded as a safer choice in the opioid group due to its relatively weak analgesic action compared to other opioids, has its risks as previously described. Prevention is the best way to treat a disease; therefore, making prescribers and opioid users aware of the risks to the infant of these actions is the first step in reducing the number of cases. CDC has recently published a guideline in order to fill in the gaps in terms of opioid prescription [5], and as a probable consequence, the number of cases has decreased in the last 3 to 4 years. Nonetheless, this number is still worryingly high [3].

Second, pregnant women are not actively submitted to exams for detection of opioid drugs, unless there is some information indicating that they may be chronic opioid users. Routine urine and meconium exams should be the norm, so that a possible addiction problem is identified and the chances of the infant having NAS are assessed. These exams are non-invasive, and are relatively quick to perform, making them an easy way to detect drug metabolites and treat the user’s addiction as well as reduce possible infant’s NAS symptoms post-partum. Adding to this, routine interviews should be performed. In a study, Romisher et al. [95] reported that nurses describe the relationship with the parents being lackluster. Nurses were seen as if holding a grudge against the parents for selfishness, irresponsibility, and feeling no remorse for others. Moreover, mothers in routine interviews also report the lack of knowledge about these problems from the nurses’ side. In addition to also being judged, they reported that their infants were treated in a different way, due to the judgments created from the fact that the mother had an addiction problem. In this study, 57% of the nurses disagreed/strongly disagreed that the mother was to blame for the infant’s symptoms, while 80% described tension between them and mothers due to the lack of involvement in the care of their infants. Despite this, 70% felt responsible for caring for the mother as well. In terms of knowledge, despite most being aware about NAS (93%), a great majority (75%) had little information on outpatient treatment.

Not only does this data show that there are still a lot of uninformed healthcare professionals on the subject, but also that standardized treatment methods are not common for NAS yet. Less than half of the participants in this study (48%) report adequate practice guidelines having been created [95]. Another identified problem was that most of the nurses thought the neonatal intensive care unit (NICU) was inappropriate for the treatment of newborns with NAS. This highlights the fact that newborns with NAS have special needs. Additionally, they refer that there is a difference between the treatment and time consumed by an infant with withdrawal symptoms, when compared to infants that are usually taken to the NICU. Consequently, most relevant issues outlined in this study were environmental issues, relationships with the mother, and inconsistencies in care. One of the most important factors that may influence these numbers is information. If both mothers and healthcare professionals are better informed, measures can be taken at earlier stages in order to prevent NAS.

## 9. Conclusions and Future Perspectives

NAS is a syndrome affecting infants born from opioid addicted mothers. One of the main causes of the increasing number of addicted pregnant women is the excess of opioid prescriptions seen nowadays. Research strategies need to take into consideration that a large number of mothers are not in a supervised maintenance program and are actually poly-drug users.

Less opioid prescriptions, earlier addiction detection, psychological support for the mothers, and a better relationship between mothers and nurses can lead to a better and more appropriate treatment for the infant. These are the goals that should be aimed to be achieved, and a lot of this can be obtained with an increased awareness of everyone involved in the process. One of the occasions where this can be properly done is during routine pregnancy appointments, where questions about this topic should be made, as well as referral to counseling. This is the first point that should be improved in the future. Concerning the detection of opioid exposure, the use of biological specimens for the detection of biomarkers is undoubtedly a valuable tool, but further studies are needed for some specimens, namely concerning the correlation of metabolites and/or biomarker’ concentrations in the different samples.

Another important factor is the treatment of both the addiction on the mother as well as NAS on the infant. A lack of standardized pharmacological treatment is seen across most literature. However, there is generally agreement concerning treating the mother’s addiction, and the most commonly used drugs are methadone and buprenorphine, with the latter showing better results in terms of safety and severity of withdrawal symptoms on the infant. In terms of NAS treatment, support therapy is usually the first choice. 

The development of guidelines and standardization of treatment worldwide is deemed necessary for improved outcomes in both the mother and the infant, and there is now plenty of information regarding opioid drugs used in the treatment of withdrawal symptoms; however, in most literature, information on the long-term effects of this type of medication is missing. Not only that, but most of the studies that exist are based in small populations. Larger studies need to be performed to assure the safety of pharmacological treatment for NAS, as well as long term studies to access its effect on the development of the infant. 

One relevant factor that seems mostly unexplored is the influence of genetics on the severity of NAS. One study from 2017 explores this possibility, and a total of seven sets of twins born from opioid addicted mothers were included. Five sets of twins born were concordant while the other 2 were non-concordant. However, as the size of this study is small, more concrete conclusions were not drawn [96]. In 2005, as previously described, a case of codeine-related death from a breastfed infant was reported. The authors discussed metabolism genetics of the mother, and since she was a fast metabolizer, codeine was quickly converted to morphine, resulting in the poisoning of the infant [17]. Genetics seems to be one of the most unexplored factors in the literature, and research around it should be done to improve treatment and better predict symptoms and pregnancy outcomes after exposure of the infant to opioids. Lastly, an innovative way of adjunctive therapy, laser acupuncture, was a plausible alternative as adjunctive therapy. 

As pointed out, healthcare professionals are seemingly misinformed and relatively unprepared to prevent and deal with opioid addicted pregnancies, as well as infants born with NAS. Therefore, guidelines and a standardization of treatment should be created in order to reduce the number of cases, and improve outcomes of opioid addiction during pregnancy. Future research must focus on assessment tool needs in order to have strong clinimetric, rather than psychometric, properties. The current lack of education of the many disciplines involved in the assessment and treatment of drug dependence during pregnancy and NAS makes it difficult for clinicians and researchers to approach this epidemic, and to avoid the potential detrimental consequences to the maternal/infant dyad. Clinicians, researchers, and government funding agencies need to combine their expertise to provide adequate education and treatment protocols for drug-dependent pregnant women and their infants with NAS. Due to this chronic relapsing disease with the potential to increase the intergenerational transmission of drug dependence and potentiate the epidemic, pregnant women and their infants will not live a full and healthy life.

## Figures and Tables

**Table 1:** Symptoms associated with Neonatal Abstinence Syndrome (NAS) and associated neurotransmitter changes.

Symptoms	Pathophysiological Mechanism
Hyperphagia	Corticotrophin increase
Hyperirritability	Dopamine decrease
Anxiety	Dopamine decrease
Diarrhea	Acetylcholine increase
Vomiting	Acetylcholine increase
Sweating	Acetylcholine increase
Hyperthermia	Noradrelanine increase
Tremors	Noradrenaline increase
Hypertension	Noradrenaline increase
Tachycardia	Noradrenaline increase
Sleep problems	Serotonin decrease

**Table 2:** Finnegan score system modified by Zimmermann-Baer et al.

Symptoms	Score
***CNS Symptoms***	
High-pitched cry	2
High pitched cry > 2 h	3
Sleeps less than 3 h after feeding	1
Sleeps less than 2 h after feeding	2
Sleeps less than 1 h after feeding	3
Mild tremors when disturbed	1
Marked tremors when disturbed	2
Increased muscle tone	2
Excoriation of skin	1
Myoclonic jerks in sleep	3
Generalized convulsion	5
***Vegetative symptoms***	
Sweating	1
Temperature 37.5–38.0 °C	1
Temperature > 38.0 °C	2
Frequent yawning	1
Mottling	1
Nasal stuffiness	2
Sneezing	1
***Gastrointestinal symptoms***	
Frantic sucking	1
Poor feeding	2
Regurgitation	2
Projectile vomiting	3
Loose stools	2
Watery stools	3
***Respiratory symptoms***	
Tachypnea > 60/min	1
Tachypnea > 60/min with retractions	2
**Total (minimum 0, maximum 37)**	
